# Role and Use of Race in Artificial Intelligence and Machine Learning Models Related to Health

**DOI:** 10.2196/73996

**Published:** 2025-07-31

**Authors:** Martin C Were, Ang Li, Bradley A Malin, Zhijun Yin, Joseph R Coco, Benjamin X Collins, Ellen Wright Clayton, Laurie L Novak, Rachele Hendricks-Sturrup, Abiodun O Oluyomi, Shilo Anders, Chao Yan

**Affiliations:** 1 Department of Medicine, Vanderbilt University Medical Center, Nashville, TN, United States; 2 Department of Biomedical Informatics, Vanderbilt University Medical Center, Suite 750, 2525 West End Ave, Nashville, TN, United States, 1 6153229374; 3 Department of Medicine, Baylor College of Medicine, Houston, TX, United States; 4 Department of Biostatistics, Vanderbilt University Medical Center, Nashville, TN, United States; 5 Department of Computer Science, Vanderbilt University, Nashville, TN, United States; 6 Department of Electrical and Computer Engineering, Vanderbilt University, Nashville, TN, United States; 7 Center for Biomedical Ethics and Society, Vanderbilt University Medical Center, Nashville, TN, United States; 8 Law School, Vanderbilt University, Nashville, TN, United States; 9 Department of Pediatrics, Vanderbilt University Medical Center, Nashville, TN, United States; 10 Duke-Robert J. Margolis Institute for Health Policy, Duke University, Durham, NC, United States; 11 Department of Anesthesiology, Vanderbilt University Medical Center, Nashville, TN, United States

**Keywords:** artificial intelligence, machine learning, race, health, ethics

## Abstract

The role and use of race within health-related artificial intelligence (AI) and machine learning (ML) models have sparked increasing attention and controversy. Despite the complexity and breadth of related issues, a robust and holistic framework to guide stakeholders in their examination and resolution remains lacking. This perspective provides a broad-based, systematic, and crosscutting landscape analysis of race-related challenges, structured around the AI and ML life cycle and framed through “points to consider” to support inquiry and decision-making.

## Introduction

The role and use of the social construct of race within health-related artificial intelligence (AI) and machine learning (ML) models have become a subject of increased attention and controversy. As noted in the National Academies’ recent report “Ending Unequal Treatment,” it is increasingly clear that race in all its complexity is a powerful predictor of unequal treatment and health care outcomes [1]. Appropriate inclusion of race within AI and ML models can identify differences in the outcomes of people with different backgrounds, creating opportunities for mitigation [2]. Yet, numerous examples exist of inappropriate inclusion of race or proxies of race in health-related models, which can harm large segments of the population [3]. For example, the long-used estimated glomerular filtration rate equation incorporated a race-based correction that overestimated kidney function in Black patients and led to delayed specialist referral and diagnosis. Similarly, the race-adjusted Vaginal Birth after Cesarean success calculator reduced predicted success rates for Black and Hispanic women, thereby discouraging trials of labor and increasing the likelihood of cesarean deliveries [3]. Such findings have informed a growing number of recommendations to remove race from AI and ML models for health in several instances [4-7]. After describing racial and ethnic differences in health care, the National Academy of Science, Engineering, and Medicine committee recommended for the Department of Health and Human Services to support elimination of interventions that exacerbate health differences and to ensure that tools and algorithms are equally valid and accurate for all people [1].

The challenge, then, is on how to achieve this goal. In recent years, statistical and computational approaches and tools have been increasingly used to identify and mitigate problems related to data representativeness and algorithmic fairness when it comes to use of race in AI and ML models [8-10]. Other bodies of work focus on characterizing what race represents within particular contexts, with an emphasis on optimizing health for all. These approaches also aim to elucidate how historical and existing social structures and practices affect health outcomes [9,11] and advocate moving from race-based to race-conscious medicine [12].

Developing and deploying both discriminative and generative AI and ML models that do justice to both computational and sociocultural aspects is challenging. Considerations of the quantitative and sociocultural factors related to race in AI and ML are complementary. Quantitative factors typically emphasize numerical model accuracy and computational techniques to enforce similar model behavior across racial groups, whereas sociocultural considerations prioritize understanding of the root causes of undesirable differences, addressing ethical and societal norms and engaging with interested parties to consider the societal impact of models. Unfortunately, the current absence of a holistic framing of this topic makes it challenging for interested and affected parties to easily and systematically interrogate and address all relevant issues that surround role and use of race in AI and ML models related to health. In fact, individuals and teams with specific expertise risk approaching this subject from a narrow perspective that fails to consider the complexities, nuances, and potential trade-offs and conflicts involved.

Comprehensive and holistic guidance on the role of race and its use in AI and ML is needed. The primary goal of this paper is to identify, frame, and examine the broad range of issues that arise. This examination is conducted across the AI and ML life cycle, identifying specific “points to consider” at each life cycle stage. These “points to consider” are meant to serve as guidance for those planning to apply this framework in practice. Recognizing that types of AI and ML projects and implementations will vary widely, the “points to consider” are framed in a manner that can be tailored to different use cases, while ensuring that key elements are addressed as part of an evaluation to ensure that each step of the process meets its goal. Issues cutting across the life cycle are also highlighted. Framing the problem in this manner can enable key interested parties, such as racial group representatives, data collectors, developers, model auditors, model users, regulatory bodies, and policy makers, to easily and comprehensively identify specific elements to examine and address for their particular use case, while being aware of the breadth of other related issues.

## AI and ML Life Cycle as a Framework to Evaluate the Role and Use of Race in Models

**Figure 1.**
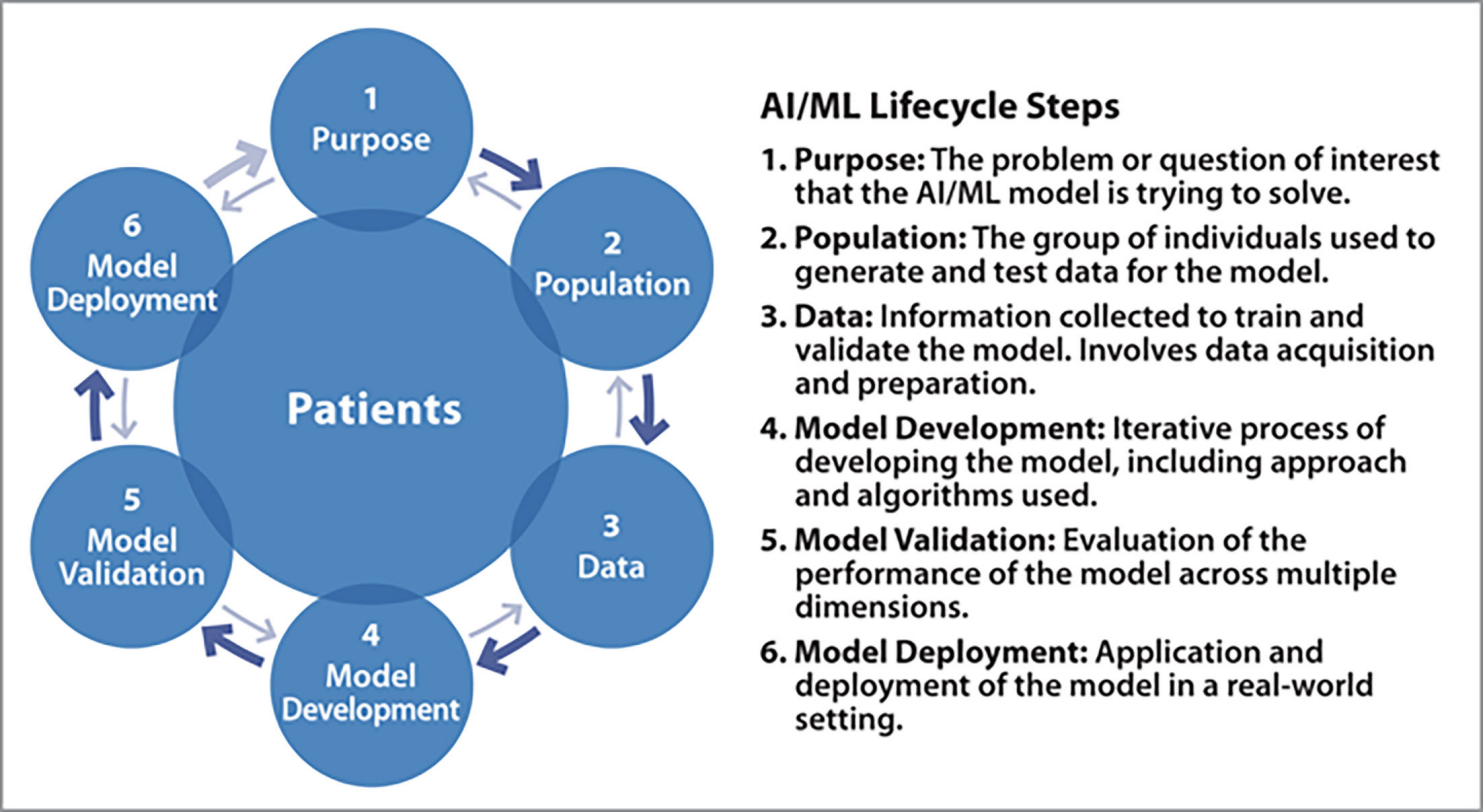
An artificial intelligence and machine learning life cycle model used to frame discussion on race. Adapted from Collins et al [17]. AI: artificial intelligence; ML: machine learning.

The AI and ML life cycle captures key steps involved in developing and implementing AI and ML models. Many variations of AI and ML life cycles have been proposed [13,14]. While the steps incorporated in such life cycles are similar, some variability exists [15,16]. In this paper, we rely on an AI and ML life cycle centered around patients to frame the discussion around role and use of race in AI and ML models for health. This life cycle has six steps, namely: (1) purpose, (2) population, (3) data, (4) model development, (5) model validation, and (6) model deployment (Figure 1). Steps in an AI and ML life cycle are interdependent, with 1 step relying on earlier ones and informing those that follow. In general, earlier steps in the life cycle influence the next step, but these connections are not necessarily unidirectional, nor are they explicitly sequential. A later step in the life cycle can affect what needs to be accomplished in earlier steps and vice versa—in Figure 1, this notion is represented by the narrower arrows flowing in the opposite direction.

The AI and ML life cycle approach provides a framework to structure and analyze issues that arise when reasoning about the role and use of race and its application in AI and ML models at each step. Notably, several of the highlighted issues and considerations in this paper are not unique to the use of race in AI and ML. As such, a broad body of work is drawn upon to inform the topic at hand, underlining the value of various perspectives. This paper focuses on a breadth of considerations with relevance to the multiple interested parties.

## Key Considerations for the Use of Race in the AI and ML Life Cycle

### Purpose

When it pertains to the use of race in AI and ML models for health, the purpose of a model could be twofold, namely: (1) a model that answers a non–race related question (eg, develop a 1-year mortality risk estimation model for all patients) but whose performance may differ across racial groups, or (b) a model that specifically evaluates a question or difference based on race (eg, examine how cancer risk factors and outcomes differ by race). In both instances, the purpose that race serves in the model must be deliberately addressed. Race, being a social construct with no biological basis, must not be conflated with genetic differences, which often reflect ancestry [18-21]. It is now well proven that race does not map to discrete genetic categories, and, as such, differences observed by race in AI and ML models should not be assumed to arise from biological differences between races [22].

AI and ML models should ideally meet the pressing needs of the target communities. In a world where some racial groups are more disadvantaged, underresourced, and have multiple unmet health care needs, the question should be asked whether the purpose of the model meets the pressing needs of the affected racial groups. Yet, approaches to systematically prioritize the needs of various groups are currently lacking. This area needs particular attention by policy makers and decision-makers to ensure that AI and ML models respond to needs and optimize outcomes for all racial groups and not just selected groups. It is also important to understand the relative risks and benefits of the AI and ML model for each racial group. While the risk-benefit equation can and should be asked throughout the life cycle, examining these early in the life cycle can identify and mitigate issues before they arise and compound in effect. Where priorities between groups conflict or compete and where risks and benefits do not match among the groups, resolution via consensus-based approaches should be used. Table 1 highlights points to consider related to race and purpose of AI and ML models.

**Table 1.** Points to consider related to race and purpose of artificial intelligence and machine learning models.

Theme	Points to consider
Genetic variation is not equal to race	Do not blindly use race as a proxy for genetic variation in models. This requires being cognizant that models evaluating human genetic variation and ancestry do not use race as a proxy for genetic variation.
Interrogate what race represents	Critically consider what race represents within a model, using findings to generate new hypotheses for examination as needed.
Prioritization of models	Consider priority of the model being developed or implemented for all affected racial groups.
Consultative approach	Gather inputs from relevant racial groups and systematically prioritize models for development and implementation that optimize benefits for all groups.
Address conflicts	Address differences in risks and benefits as well as conflicts in interests between groups.

### Population

Population in Figure 1 represents all categories of patients, research participants, community members, and other individuals from whom data are generated and used to train and test AI and ML models. Unfortunately, categorizing subsets of the population into racial groups can lead to misrepresentations and misconceptions when used within AI and ML models. Two common misconceptions are that discrete race categories carry the same meaning across countries and that they remain unchanged over time. Yet, definitions of racial categories can vary within and among countries [23-25]. Furthermore, these definitions have historically changed over time, including the recent reclassifications by the Office of Management and Budget in the United States that introduced a new race category of “Middle Eastern or North African,” among other changes [26,27]. Individuals who do not self-identify with a single or any race category also add complexity [28,29].

Those from whom data are used in creating AI and ML models and on whom the models are implemented are not passive bystanders but rather are interested parties who directly experience the risks and benefits of developed models. Given the lack of public understanding of these, clear and proven community engagement strategies and collaborative partnerships that build trust must be used before, during, and after implementation of AI and ML models [30,31]. For those who have less familiarity with these tools, this may require selecting appropriate community representatives to ensure that these groups have a voice and provide inputs into the process—akin to what is done in some consent scenarios [32,33]. As the target population may have important insights into what is at stake, these engagements can help optimize mutual benefits and reduce disproportionate risks for particular racial groups throughout the model’s development and deployment phases. Capacity-building initiatives will help these groups better understand what is at stake as related to AI and ML models, support informed participation and sharing of data by these groups, and allow the groups to engage in highlighting areas where models do not apply accurately to them [34].

Investigators from groups that have been less included in research can also provide valuable insights into the development and use of AI and ML. An example of such a capacity building and workforce development initiative is the Artificial Intelligence/Machine Learning Consortium to Advance Health Equity and Researcher Diversity that aims to increase participation and engagement of researchers and communities from all backgrounds in AI and ML initiatives [30]. Table 2 highlights points to consider around race and populations on whom models are developed and implemented.

**Table 2.** Points to consider around race and populations on whom artificial intelligence (AI) and machine learning (ML) models are developed.

Theme	Points to consider
Meaning of racial categories	Understand what various categories of race mean in the context of the model to be developed and whether these definitions have changed over time.
Generalizability of racial categories	Examine generalizability of the racial categories used in developing the model, especially whether these categories apply similarly in different locations, countries, and time periods.
Engagement and collaborative partnership	Use appropriate community engagement and collaborative partnership strategies to inform all relevant stages of model development and to build trust.
Build capacity to comprehend AI and ML	Build capacity among all racial groups to understand the role of AI and ML as well as specific relevant models and their implications.

### Data

The quality and quantity of the training data provided to a machine learning model have a major impact on its performance, such that inadequacies in the data can undermine the applicability of resulting models [35]. Incomplete or skewed collection of data from different populations can lead to flawed tools. The challenges of using nonrepresentative data for racial groups have been broadly reported. An often-cited example is that of pulse oximetry devices that have been shown to perform worse for Black patients than for White patients—largely because these devices were trained on data from mostly White patients [36-38]. Even when various racial groups are represented in the data, the quality of their data, from the perspective of completeness, correctness, and freshness, often varies. As an example, in the United States, data about race and ethnicity are more likely to be incorrect for non-White patients in administrative databases [39,40]. The proportion of missing racial data can also vary widely between racial groups within the same dataset [40]. In addition, the quality can be influenced by whether information is self-reported or recorded by observers (eg, health care providers) [40-42]. How data are labeled, including when automated approaches are used, can also introduce bias that adversely impacts certain racial groups [43,44]. Beyond issues originating from data themselves, inappropriate use of available data in model development (eg, using medical costs as a proxy of a patient’s health need for resources) can lead to consequences detrimental to certain subpopulations [45].

Often, the differences observed between racial groups reflect other unaccounted factors such as social, economic, and environmental influences [46,47]. This notion is demonstrated in a study in which the prognostic performance of the introduced model for predicting in-hospital mortality for Black patients improved when other nonmedical drivers of health (NMDoH), such as location, income, wealth, language, and education, were added into the model [48]. Other studies have shown that adding NMDoH data to AI and ML models can help reduce errors in outputs and provide insights into some of the associated factors contributing to differences by race [48-52]. The question should therefore always be asked about whether NMDoH data can be used to augment or replace race in models [53-55]. In addition, incorporating genetic (ancestry) and other biological data when available can further improve models that might consider using race data [19,28].

Given existing challenges around completeness and quality of race-based data within datasets, it is often necessary to ensure appropriate data collection and preprocessing approaches [56]. Beyond working toward the collection of more complete and representative data, statistical and computational approaches can be used to recognize and, at times, mitigate data-related deficiencies. Common mitigation approaches related to data include (1) removing race information from training data [8,56-58], (2) adding relevant information as new variables [59,60], (3) reweighting or rebalancing [61], (4) removing disparate impact [62], (5) learning fair representations [63], and (6) developing or augmenting with synthetic data [64]. It should be recognized that simply discarding race from the equation can sometimes lead to greater harm [65]. A general guideline is to include race as a variable only when it can enhance model fairness and when there is a clear understanding of its role and meaning within the datasets. It is also important to note that no single approach will best improve fairness in all cases. Therefore, determining which data preprocessing approaches should be used will depend on the particular AI and ML use case, ideally informed by individuals or teams with relevant expertise and by comprehensive evaluation obtained in subsequent stages of the life cycle. Table 3 outlines key pros and cons of each of these approaches.

**Table 3.** Common data preprocessing approaches for mitigating racial bias in artificial intelligence and machine learning models.

Approach	Description	Pros	Cons
Remove race information [8,56-58]	Discard race as a variable from models to be developed.	Can prevent the perpetuation of race-based medicine that negatively impacts underserved subpopulations.	Blindly and solely relying on this strategy (ie, “fairness through unawareness”) might negatively impact fairness when race correlates with unaccounted critical variations in health outcomes.
Add relevant information as new variables [59,60]	Collect and incorporate important variables such as NMDoH^a^ and relevant biological indicators or measures.	Can oftentimes help explain variations in patients’ outcomes. Can mitigate or remove the independent impact of race in model outcomes.	Might create redundancy or induce noise if new variables carry invalid information.
Rebalance or reweigh existing data [61]	Randomly oversample underrepresented racial groups or put more weight on these groups.	Balance representativeness and prevent majority domination in model training. Low computational cost.	No new information is introduced. Can cause overfitting and undermine generalizability.
Mitigate variable distinguishability [62]	Adjust the values of individual variables to make the relevant distributions across racial groups less distinguishable.	Can effectively mitigate bias related to disparate impact.	Can oversimplify complex relationships in the data. Might lose critical clinical information. Can reduce the overall accuracy. Might not generalize to other cohorts.
Learning fair representations [63]	Learn a latent representation for each data instance that obfuscates information about race.	Can effectively mitigate differences in model performance related to disparate impact.	Might lose critical clinical information. Can reduce the overall accuracy. Might not generalize to other cohorts. Can create difficulties for model troubleshooting.
Develop synthetic data [64]	Generate unseen data conditioned on protected attributes (eg, race) and merge with real data for model training.	Can enhance the representativeness of racial groups that are not well represented in the data. Might improve fairness and overall model accuracy simultaneously.	Synthetic data may not fully represent the complexity of specific use cases. Can amplify model performance differences in real data when inappropriately generated. Data creation can be resource-intensive.

a NMDoH: nonmedical drivers of health.

Mechanisms should be set in place to highlight the provenance (origin and history) and lineage (path taken from original state to current state) of the race data used in AI and ML model [66,67]. This will help users evaluate the quality and integrity of the data for the AI and ML model. Moreover, it can reveal whether the data were obtained ethically and comply with regulatory guidelines.

Use of the dataset “nutrition labels,” in particular, is increasingly being advocated. The dataset nutrition labels aim to establish standardized metadata that highlight the key ingredients of a dataset as well as unique or anomalous variables regarding distribution, missing data, and comparison with other “ground truth” datasets [68]. Labels related to race should detail the characteristics of different racial groups within a cohort. To support implementation of provenance and lineage of datasets, projects can leverage available metadata and data lineage tools [66]. Table 4 summarizes key points to consider around data in informing use and role of race within AI and ML models for health.

**Table 4.** Points to consider regarding race and the data used in artificial intelligence and machine learning models.

Theme	Points to consider
Reliability of data source	Determine the reliability of the data sources from which the racial data are derived.
Representativeness of data	Assess whether data for all relevant racial categories are adequately represented to train the model and, if not, assess the feasibility of collecting more data for underrepresented subgroups.
Data labeling	Evaluate the degree to which the race-based data were appropriately labeled.
Data preprocessing	Apply appropriate approaches to handle data quality issues and to preprocess the data (Table 3).
Data provenance and lineage	Gather and use provenance and lineage information on the data.

### Model Development

In addition to the characteristics of the data underlying models, inappropriate outcomes of health-related AI and ML can also arise from the architectural design of the model [55,69]. To address both data and model challenges, a large number of approaches have been developed to enhance data and model quality during the model development stage [8,64,69-71]. These approaches acknowledge that algorithms are not impartial and that certain design choices by their architects can lead to better results in mitigating and addressing racial bias. Common types of algorithmic fairness include individual fairness (ie, individual patients with similar data have similar likelihood of benefiting from the model), counterfactual fairness (ie, the patient-level model outcomes are unaffected by variations in protected attributes such as race and other demographic information), and group fairness (ie, model outcomes are similar across groups of sensitive attributes) [72].

Pertaining to race, group fairness is particularly relevant, given its use in exploring the adequacy of application across demographic groups. Group fairness aims to define, quantify, and mitigate unfairness from AI and ML models that may cause disproportionate harm to certain subpopulations, such as to specific racial groups [73]. Numerous definitions of group fairness exist, each corresponding to a quantitative fairness metric that emphasizes a specific concern. Thus, the selection of fairness metrics should be based on the specific needs of each use case, recognizing that all metrics cannot be achieved at the same time [74]. Fairness metrics can be enforced during, as well as after, model training through the addition of nondiscrimination constraints as part of the objective function [69]. While enforcing metrics can induce models that are more generalizable, the effectiveness of such approaches can vary and they could impact the overall model accuracy and introduce a higher level of complexity and cost for model implementation [70,75,76]. Moreover, enforcing fairness for one sensitive attribute (or one fairness metric) can inadvertently lead to unfair outcomes for another sensitive attribute (or another metric). As such, selection of the fairness enforcement strategy, including whether there is a need to do so, should be thoroughly assessed and tailored to specific use cases.

Tensions between algorithmic fairness and model accuracy can arise during model training. In such situations, it will be necessary for stakeholders to be engaged to determine context-specific and acceptable trade-offs aligned with ethical and clinical priorities. At the same time, emerging evidence on strategies highlighted in the “Data” subsection, such as synthetic data augmentation and the collection of more representative and cleaner datasets, demonstrates the potential to simultaneously improve performance and reduce fairness disparities [77-79]. However, when and how best to apply these strategies require further investigations.

A subset of available data needs to be set aside, using strategies such as stratification and temporal selection, to conduct an initial evaluation of the model’s accuracy and applicability across groups to provide feedback on the effectiveness of considered approaches for improving fairness. It should be noted, however, that directly applying these approaches can risk masking rather than resolving the deeper systemic issues that cause problematic applications, such as unequal access to health care or race-based patient treatment.

Given that race may correlate with social, environmental, and economic factors, appropriate approaches must be implemented during model development to handle such correlations when race is used as a covariate. At the very least, differences observed by race in AI and ML models should be scrutinized to better understand the exact causes of the observed differences, which may involve other NMDoH. These observed differences should trigger hypotheses with subsequent examination to better understand the causes. Examination of variations within racial groups (within-group designs), using techniques such as hierarchical models, can provide insights into the causes of observed differences [80,81]. Furthermore, when differences between racial groups are detected in models, a systematic approach should be applied to reduce differences between the groups in a unified model, while being attentive to not compromising performance [82]. However, if model performance is significantly affected in the unified model, it will be necessary to evaluate the implications of using different models by race or whether to consider other variables. Finally, attention should also be paid to whether models leverage embedded demographic information (such as race) as shortcuts to make predictions, even when race is not explicitly included as a variable [83]. Benefits of eliminating these demographic shortcuts and approaches to use will depend on the particular case. Table 5 highlights points to consider during model development.

**Table 5.** Points to consider regarding race during artificial intelligence and machine learning model development.

Theme	Points to consider
Fairness definition	Determine the fairness definitions and corresponding metrics to pursue for the current use case.
Model selection and optimization	Ensure that the selected model and optimization algorithm do not deliver outputs that treat some groups inappropriately.
Assess for fairness	Before using any fairness enforcement approaches, determine whether the trained models are unfair among racial groups (subgroup analysis) and identify the reasons for the observed unfairness.
Enforce fairness	Compare and optimize fairness enforcement approaches in the model development stage.
Examine causes of differences	Critically examine the various possible causes of difference by race in order to prevent inappropriate application of models.
Within-group analysis	Perform within-group analyses.
Evaluate the impact of fairness enforcement	Assess the impact of model fairness enforcement approaches on both fairness and model performance.
Unified versus distinct models	When model performance for certain racial groups is unacceptably sacrificed for achieving fairness through a unified model, assess the ethical and technological feasibility of developing distinct models for different racial groups that can break out the tension between performance and fairness.
Embedded race information	Determine whether the model uses embedded race information as shortcuts for factors such as NMDoH^a^ in decision-making, and the implications of eliminating such shortcuts to best meet use case for the model.

a NMDoH: nonmedical drivers of health.

### Validation and Assessment

Rigorous validation of model behavior should be conducted to ensure that the model performs as expected before deployment to ensure generalizability. This model validation and testing should be performed for both model performance and fairness across various scenarios, populations, and under as many different constraints as possible. This is because the real-world environment in which the developed model will be deployed might differ from the data generation environment used during the model’s development. While it is not uncommon for the performance of a model to deteriorate from what was observed during development, recent findings have shown that the level of model performance achieved in a development dataset does not necessarily transfer to different datasets or application settings [84]. Examples of such discrepancies include variations or inconsistencies in (1) the demographics, NMDoH, and clinical characteristics of patient cohorts; (2) the availability of variables; (3) measurement techniques such as medical devices and their algorithms; (4) clinical care protocols; and (5) data collection and labeling procedures.

Models developed in one region or country might not translate to another without proper modifications. Considering all these complexities, implementing a silent-mode predeployment validation, which mimics site-specific settings without showing results to end users [14], could be the optimal strategy for ensuring the robustness and effectiveness of the model before it goes live [84]. Ideally, additional measures beyond performance and algorithmic fairness, such as the impacts on care quality, eligibility, cost, and outcomes, should be thoroughly assessed across the various racial groups as part of predeployment assessment [85,86]. The cost-benefit ratio of different AI and ML interventions becomes particularly relevant, given the close connection of race with differences in health-related outcomes across racial groups. In particular, the cost-benefit of an AI and ML model should be compared against other models, as well as against other proven interventions and approaches to inform which model should be considered for use relative to alternative interventions. Model assessment should also incorporate the feasibility of adoption, given the multiple infrastructure, financial, and human resource constraints faced by various populations and settings. It might not be justifiable to advocate for deploying models that are too costly to deploy to groups with limited resources without requisite measures to assure success in implementation and outcomes. Table 6 summarizes key considerations surrounding validation and assessment of models.

**Table 6.** Points to consider regarding predeployment assessment of artificial intelligence and machine learning models.

Theme	Points to consider
Preimplementation testing	Conduct rigorous testing on model performance and fairness on previously unseen data before deployment.
Outcomes and risk assessments	Assess whether the impacts of the model on outcomes and risk allocation are acceptable.
Feasibility assessment	Conduct feasibility assessments on implementation success by sorting out the disparities associated with race.
Cost-benefit evaluation	Examine cost-benefit analysis results of the model.
Comparative cost-benefit	Compare the cost-benefit of the model against other proven interventions.

### Model Deployment

All implemented AI and ML models should be audited prior to deployment and monitored once deployed [87]. Even when a model does not have a race variable, it can still generate unfair outcomes because of potential correlations between race and other variables. Efforts to improve explainability of AI [88-90] can support decision-making on which AI and ML models an organization should deploy [91,92]. Of particular relevance are external audits of algorithms, which often require deploying organizations to work closely with model developers [93,94]. Continuous monitoring of deployed models is essential, given that data and model drift can have significant impact on model performance and fairness across groups. By using processes and methods to detect drift, organizations can identify models that need updating or discontinuation [95]. Like other informatics-based interventions, AI and ML models can have unintended consequences, which must be monitored and mitigated using various available approaches [96-98]. Unintended consequences can further be ameliorated through awareness of the interactions between model outputs and the users of the model. This will reduce model outputs from being incorrectly interpreted by the users who often have their outlook.

Deliberate application of principles to assure optimal outcomes for all can further uncover and mitigate negative impacts of AI and ML models that incorporate race. Well-accepted approaches, such as those by Whitehead and Dahlgreen [99], are particularly applicable and can be adopted for AI and ML models being deployed. These would include a requirement for AI and ML models to (1) level up, and not level down; (2) improve the status of those who are disadvantaged; (3) narrow the health divide; (4) reduce social inequities throughout the whole population; (5) tackle the fundamental social determinants of health; and (6) facilitate equal access to services and ensure that particular racial groups do not pay more to access the tools than others [99]. As appropriate, distributive justice approaches that emphasize allowing all people to achieve their optimal health and resource allocation across the various racial groups should also be used [52]. Table 7 summarizes key considerations in deploying models when race is considered.

**Table 7.** Points to consider regarding race and deployment of artificial intelligence (AI) and machine learning (ML) models.

Theme	Points to consider
Deployment context	Ensure context within which the model is being deployed is appropriate for that model.
Site-specific model assessment	Evaluate performance of the model for various groups within the specific deployment setting.
External model audit	Models need to be independently audited prior to deployment.
Monitor data and model drift	Implemented models should be monitored to detect performance changes and to inform updates needed or need for model discontinuation.
User awareness	Maintain vigilance on how users interact with models and interpret the model’s outputs.
Unintended consequences	Monitor and mitigate unintended consequences.
Outcomes for all	Use accepted frameworks to evaluate impacts of the AI and ML model on optimal access to health care for all.

### Crosscutting Considerations

In addition to issues arising at each stage of the life cycle, there are several crosscutting issues regarding the role and use of race across the AI and ML life cycle that deserve particular attention.

#### Teams

Teams with different types of expertise are involved at the various stages of the AI and ML life cycle. As pertains to models that involve patients with multiple races, individuals with various backgrounds in teams can bring different and relevant insights and perspectives at each stage. Beyond community engagement and engagement with community representatives, deliberate capacity building and involvement of individuals with diverse backgrounds are also relevant for developers and implementer teams of these models. Teams also need to bridge computational and social-cultural aspects of model development and implementation by incorporating multidisciplinary team members. This multidisciplinary approach was used in the authorship of this paper in recognition of the value of different perspectives.

#### Governance

Governance mechanisms that ensure that data are obtained and used ethically and approaches for the adoption and monitoring of race-based AI and ML models must be in place. Unlike medicines and devices that are often tightly regulated, regulation of AI and ML models is nascent at best [100], but the pervasiveness of race-biased predictive models in broad use calls for extra vigilance. This necessitates robust governance mechanisms, especially when AI and ML models can variably impact the various racial groups [45,101].

#### Organizational Capabilities

Institutions that serve disadvantaged groups are less likely to have the organizational capabilities to develop, implement, and monitor AI and ML models and applications [102]. Costs across the AI and ML life cycle are often prohibitive, which can impede development and use when requisite human, financial, and infrastructure resources are unavailable. Understanding and narrowing resource and capability gaps across institutions will help ensure that AI and ML benefits are derived by all groups.

#### Evaluation

To assure high-quality models, evaluation must be incorporated at every step in the life cycle. Evaluations across the life cycle can range from adequacy of community engagement strategies, quality assessment of data, evaluations performance of the model, model generalizability, impacts on health outcomes, ethical considerations, cost-benefit, and acceptability to those affected, among others. These evaluations can uncover gaps and inform mitigation strategies.

## Conclusions

The role and use of race in AI and ML models for health will continue to elicit debate and are deserving of further research and examination. At the very least, caution must be exercised when considering issues surrounding role and use of race within AI and ML models or in interpreting differences in model outputs based on race. This work provides broad-based guidance to those wrestling with this topic at any of the stages of the AI and ML life cycle and should stimulate renewed and comprehensive scrutiny on role and uses of race within AI and ML models for health. The proposed framework will need to be applied to real-world case studies to evaluate its use. Further work is also needed to address the tension between model accuracy and fairness.

## Abbreviations

AI: artificial intelligence
ML: machine learning
NMDoH: nonmedical drivers of health
